# Patch-based models and algorithms for image denoising: a comparative review between patch-based images denoising methods for additive noise reduction

**DOI:** 10.1186/s13640-017-0203-4

**Published:** 2017-08-24

**Authors:** Monagi H. Alkinani, Mahmoud R. El-Sakka

**Affiliations:** 1 Department of Computer Science, University of Jeddah, Asfan Road, 285, Dhahban, Jeddah, 23881 Saudi Arabia; 2 Department of Computer Science, Middlesex College, Western University, 1151 Richmond Street, London, Ontario, N6A 5B7 Canada

**Keywords:** Patch-based image denoising, Bilateral filter, Non-local means filtering, Probabilistic patch-based filtering, Dictionary learning filtering, K-SVD, Gaussian patch-PCA filtering, BM3D

## Abstract

**Background:**

Digital images are captured using sensors during the data acquisition phase, where they are often contaminated by noise (an undesired random signal). Such noise can also be produced during transmission or by poor-quality lossy image compression. Reducing the noise and enhancing the images are considered the central process to all other digital image processing tasks. The improvement in the performance of image denoising methods would contribute greatly on the results of other image processing techniques. Patch-based denoising methods recently have merged as the state-of-the-art denoising approaches for various additive noise levels. In this work, the use of the state-of-the-art patch-based denoising methods for additive noise reduction is investigated. Various types of image datasets are addressed to conduct this study.

**Methods:**

We first explain the type of noise in digital images and discuss various image denoising approaches, with a focus on patch-based denoising methods. Then, we experimentally evaluate both quantitatively and qualitatively the patch-based denoising methods. The patch-based image denoising methods are analyzed in terms of quality and computational time.

**Results:**

Despite the sophistication of patch-based image denoising approaches, most patch-based image denoising methods outperform the rest. Fast patch similarity measurements produce fast patch-based image denoising methods.

**Conclusion:**

Patch-based image denoising approaches can effectively reduce noise and enhance images. Patch-based image denoising approach is the state-of-the-art image denoising approach.

## Review

### Introduction

**Fig. 1:**
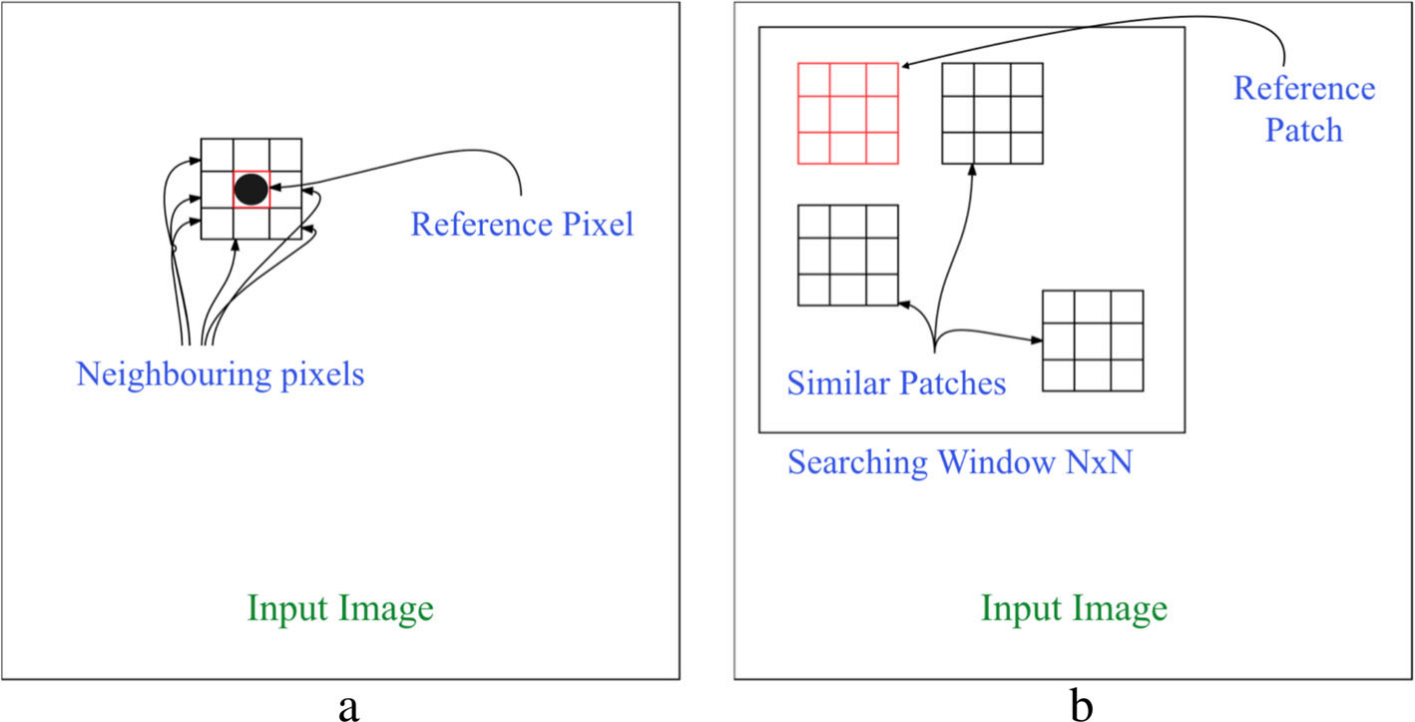
Image denoising approaches: **a** filtering based on neighboring pixels located within a kernel in pixel-based denoising schemes and **b** filtering based on patches located within a search window in patch-based denoising schemes

The noise level in digital images may vary from being almost imperceptible to being very noticeable. Image denoising techniques attempt to produce a new image that has less noise, i.e., closer to the original noise-free image. Image denoising techniques can be grouped into two main approaches: pixel-based image filtering and patch-based image filtering. A pixel-based image filtering scheme is mainly a proximity operation used for manipulating one pixel at a time (pixel-wise) based on its spatial neighboring pixels located within a kernel. On the other hand, in patch-based image filtering, the noisy image is divided into patches, or “blocks,” which are then manipulated separately in order to provide an estimate of the true pixel values (patch-wise) based on similar patches located within a search window. This approach utilizes the redundancy and the similarity among the various parts of the input image. Figure 1 shows the mechanism of the two approaches.

It is now common in image denoising field to utilize patch-based models and algorithms instead of pixel-based approaches to produce most promising estimate of the noise-free images. However, there are both advantages and disadvantages in the use of patch-based models and algorithms. There are several advantages of patch-based approaches. Smoothing flat regions is the most important aspect. Redundancy between patches enable patch-based approaches to properly smooth flat reigns. A second advantage of using patch-based models and algorithms approaches is that it can preserve fine image details and sharp edges. However, there could be some disadvantages for patch-based models and algorithms. First, although similarity between patches assists in estimating flat regions, so is the averaging. It is, therefore, quite time-consuming to group and compare similar patches. This might mean that each patch has multiple estimates and patches are overlapped. Secondly, while it may be that patterns and textures seem clear with less noise, patch-based models and algorithms usually exploit large number of parameters, which can be extremely difficult to adjust properly. We believe that the advantages of patch-based methods far outweigh their disadvantages, as modern computers are significantly fast, and have large memory spaces.

In this work, the patch-based image denoising schemes are analyzed from two different aspects: (1) the performance of patch-based denoising techniques in terms of image denoising quality and (2) the performance of patch-based denoising techniques in terms of computational time, where various patch-based denoising techniques are addressed.

A literature survey was conducted to evaluate the most recent patch-based denoising improvements for additive noise. Following the literature survey, there is an empirical study, which is used to evaluate the performance of various patch-based denoising techniques in terms of their accuracy and run times at various noise levels.

## Patch-based image filtering

In patch-based denoising techniques, the input noisy image is divided into patches (i.e., blocks). The blocks are then manipulated separately in order to provide an estimate of the true pixel values. In this section, various patch-based image denoising algorithms are presented and their efficiency with respect to image denoising are studied.

### Averaging patch-based: non-local means

**Fig. 2:**
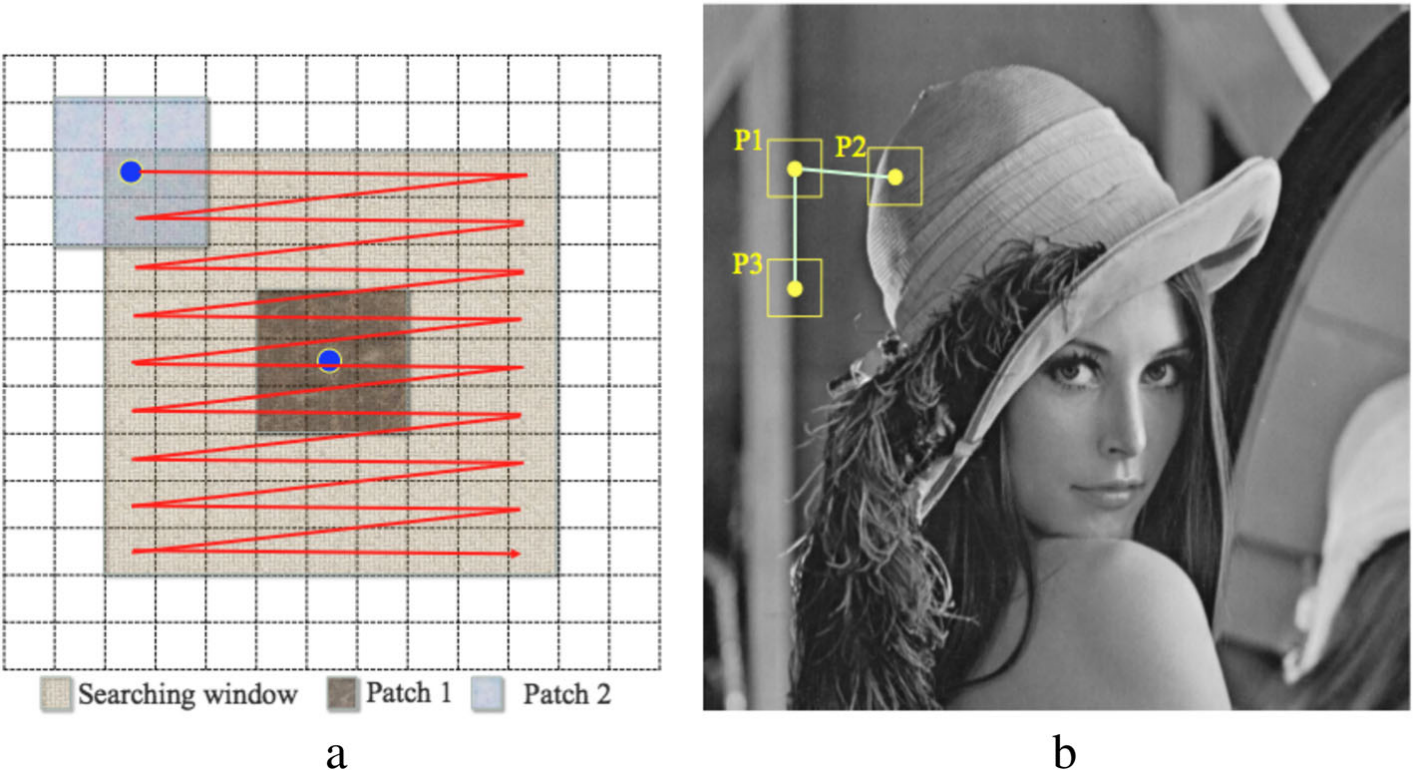
Similarity between patches. **a** NL-Means patches as a raster scan in a search window. **b** Patch P3 is similar to P1 more than patch P2; hence, P3 will get a weight larger than P2 weight

Non-local means (NL-Means) is a patch-based filter proposed by Buades et al. [6] as a modification to the pixel-wise bilateral filter [4, 16, 38, 50, 58, 61]. Like the bilateral filter, the NL-Means filter blurs the homogeneous areas and preserves edges. The NL-Means filter divides the input image into sub-images and then filters each sub-image separately in a technique that is referred to as being patch-wise. Each sub-image contains several patches. As in the bilateral filter, similarity is measured based on two measurements: (1) the Euclidean distance between the centers of the patches and (2) the luminance distance between the patches. In contrast to the bilateral filter, patches are compared within a search window instead of with the pixels of the neighbors. This is why it is called a non-local method. Patches with similar gray levels have larger weights when they are averaged. Figure 2 a shows the NL-Means patches and how to find the similar patches in a raster scan in a search window. Figure 2 b illustrates the fact that patches with a similar gray level, for example, P1 and P3, should be assigned a larger weight than that to be assigned to P2. The edges in NL-Means filtering are preserved regardless of their direction.

The estimated value NL-Means [ *v*]_*i*_, for a pixel *i*, is computed as:

1 $$ \text{NL-Means}[\!v]_{i}=\sum\limits_{j\in I}\omega(i,j)[\!v]_{j},  $$

where [ *v*]_*i*_ and [ *v*]_*j*_ are pixel intensities at locations *i* and *j*, respectively, and *ω*(*i*,*j*) is a similarity measure between the pixels *i* and *j*. The similarity measure weight satisfies the condition 0≤*ω*(*i*,*j*)≤1 and $\sum _{j}\omega (j,i)=1$. The similarity weight depends on the gray level similarity and the Euclidean distance between vectors *N*[ *v*]_*i*_ and *N*[ *v*]_*j*_, where *N*[ *v*]_*k*_ denotes a square neighborhood of fixed size and centered at a pixel *k*. The weights are described as:

2 $$ \omega(i,j)=\frac{1}{Z(i)}e^{-\frac{\parallel\left(N[v]_{i}\right)-\left(N[v]_{j}\right)\parallel^{2}}{h^{2}}},  $$

where *Z*(*i*) is a normalization factor and *h* is a filtering parameter set depending on the noise level.

The level of noise determines the sizes needed for the search window and patches. For a robust comparison between patches, the size of the patches increases when the noise level is high. Accordingly, the value of the filtering parameter *h* increases as the size of the patch is increased. Meanwhile, the size of the search window must be increased in order to find more similar patches.

NL-Means filter is considered the cornerstone of many patch-based denoising methods. It can be adapted easily to many other applications, such as, multi-view image denoising. Nevertheless, there are some disadvantages of NL-Means filter. The filter is computationally expensive due to the large amount of weight computations between similar patches. Another disadvantage is that the NL-Means filter is a spatial domain filter though convolution can be easily implemented in the frequency domain.

NL-Means filter has too many modifications. Improving the way of assigning the weights between patches would improve the performance of the NL-Means method. Hedjam et al. [25] improved the process of adjusting the weights in the NL-Means by using Markovian clustering. Wu et al. [63] used a statistical shrinkage perspective when assigning the weights in NL-Means via using James-Stein [30] shrinkage estimator. Lai and Dou [35] introduced an improved neighborhood pre-classification strategy for optimized weight kernels of NL-Means filter. Khan and El-Sakka [32] introduced a variant of the NL-Means scheme by using a thresholding step to reduce the number of similar patches before weight averaging the patches.

NL-Means is a spatial domain filter, transferring this filter to the frequency domain would help in suppressing more noisy signal. Enríquez and Ponomaryov [17] transferred the patches of the NL-Means to the frequency domain and used discrete cosine transform with a threshold to estimate true patches. Zhong et al. [69] combined the NL-Means with Lee filter [36] for SAR image enhancing. Chan et al. [7] incorporated a median filtering operation indirectly in the NL-Means method for denoising low signal noise ratio (SNR) images. Maruf and El-Sakka [43] projected NL-Means patches into a global feature space before performing a statistical *t* test to reduce the dimensionality of this feature space. They gathered similar patches globally. Irrera et al. [28] adapted NL-Means for denoising X-ray images (XNL-Means), and then they applied an additional multi-scale contrast enhancement in the frequency domains.

NL-Means filter could be adapted to improve other image processing applications (e.g., segmentation, recognition, and video denoising). Zhan et al. [66] introduced an extension to the NL-Means method for ultrasonic speckle reduction. They assigned the patch similarity weights iteratively in a lower dimensional subspace using principal component analysis (PCA). Xu et al. [65] adapted the NL-Means to be use for microscopy cell images via a frequency transform. Genin et al. [20] adapted a modified version of the NL-Means filter for detecting small objects by background suppression. Background pixels are estimated by a weighted average depending on the similarity between neighborhoods pixels. Kim et al. [33] adapted the NL-Means filter for noise reduction and enhancement of extremely low-light video. They use a motion adaptive temporal filter using gamma correction with adaptive thresholds before the NL-Means filter. Xu et al. [64] adapted the idea of patching from the NL-Means for filtering polarimetric synthetic aperture radar (POL-SAR) images; they use simultaneous sparse coding for transferring the patches into the frequency domain before assigning the weights.

### Probabilistic patch-based filter

The probabilistic patch-based (PPB) filter, which works in the spatial domain, was proposed by Deledalle et al. [12] as an extension of the NL-Means filter. The PPB approach is one of a few denoising techniques that can provide a general denoising methodology for various noise models. Thus, it is more general than the NL-Means and can be applied where there is additive noise or multiplicative speckle noise. The PPB filter is a statistically based similarity scheme that depends on the distribution model of the noise. The weighted average is used for the Gaussian noise distribution in the NL-Means, but the PPB filter applies smoothing based on the maximum likelihood estimator (MLE). The PPB is expressed as a weighted maximum likelihood estimation (WMLE) problem. The weight is derived from the data by improving the isotropy of the filter—non-iterative probabilistic patch-based filter (Non-iPPB)—and it can be iteratively defined based on the similarity of the patches—iterative probabilistic patch-based filter (It-PPB).

#### Weighted maximum likelihood estimator

Using a weighted maximum likelihood estimation for image denoising is not a new technique; it was first used for image denoising by Polzehl and Spokoiny [51, 52]. PPB redefined the weights in term of a patch-based approach. PPB image denoising is considered to be an estimation $\hat {u}$ of the true image *u* which originates from the noisy image *υ*. The images are defined over a discrete regular grid *Ω*. A pixel value is described as *i*, and its neighbor is *j* at the location (*x*,*y*)∈*Ω*. The noise model is considered as being defined by the parametric noise distribution “likelihood” $p(i\mid \theta _{i}^{*})$, where $\theta _{i}^{*}$ is an unknown space-varying parameter. The denoising of an image is equivalent to finding the best estimation $\hat {\theta }_{i}$ for $\theta _{i}^{*}$ for all pixels. The MLE at each location (*x*,*y*) estimates $\hat {\theta }_{i}$ from a set $S_{\theta _{i}^{*}}$ of the distributed random variables around it by:

3 $$\begin{array}{@{}rcl@{}} \hat{\theta}_{i} & \overset{\bigtriangleup}{=} & \arg\underset{\theta_{i}}{\max}\underset{j\in S_{\theta_{i}^{*}}}{\sum}\log p\left(j\mid\theta_{i}\right),\\ & \overset{\bigtriangleup}{=} & \arg\underset{\theta_{i}}{\max}\underset{j}{\sum}\delta_{S_{\theta_{i}^{*}}}(j)\log p\left(j\mid\theta_{i}\right), \end{array} $$

where $\delta _{S_{\theta _{i}^{*}}}$ is an indicator function for $S_{\theta _{i}^{*}}$ (i.e., $\delta _{S_{\theta _{i}^{*}}}=1$ if $j\in S_{\theta _{i}^{*}}$ or 0 otherwise). The indicator function has been derived from the data as weights *ω*(*i*,*j*)≥0 in Polzehl and Spokoiny [52] and Polzehl and Tabelow [51], where it is used as weight for adaptive pixel-wise filters. However, the indicator function in PPB is used as weight function to form the WMLE:

4 $$ \hat{\theta}_{i}\overset{\bigtriangleup}{=}\arg\underset{\theta_{i}}{\max}\underset{j}{\sum}\omega(i,j)\log p(j\mid\theta_{i}).  $$

#### Defining the weight between patches

In Subsection 2.1, the weights in the non-local means filter are defined by comparing the similarities of the two patches [ *v*]_*i*_ and [ *v*]_*j*_ centered around the two locations *i* and *j*, respectively. A weighted Euclidian distance between the two patches defines the level of similarity. The objective of the PPB filter is to generalize and extend the idea of the Euclidean distance weight used in the non-local means filter so that it can be adapted to non-additive noise models. The weights used in the probabilistic patch-based method are estimated using the probability of the two patches in a noisy image having the same parameters. By following the same idea as the weight in the non-local means filter and assuming equal values for *i* and *j* in the two statically similar patches [ *v*]_*i*_ and [ *v*]_*j*_, PPB weights would be defined as:

5 $$ \omega(i,j)^{(\text{PPB})}\overset{\bigtriangleup}{=}p\left(\theta_{[v]_{i}}^{*}=\theta_{[v]_{j}}^{*}\mid\upsilon\right)^{\nicefrac{1}{h}},  $$

where $\theta _{[v]_{i}}^{*}$ and $\theta _{[v]_{j}}^{*}$ are the patches extracted from the image *θ* ^∗^ and *h* is larger than 0, which indicates the size of the patch in PPB. The *h* acts as *σ* in the NL-Means algorithm in order to control the filtering amount. The probability of the similarity of the patches pixels is decomposed into a product of the probabilities of *k* neighbors: $\prod _{k}p\left (\theta _{i,k}^{*}=\theta _{j,k}^{*}\mid \upsilon _{i,k},\upsilon _{j,k}\right)$.

#### Iterative WMLE denoising

In order to improve the performance of the PPB algorithm, the probability of a similarity is estimated iteratively. The weight, at each iteration, is expressed as the product of two terms: (1) the probability of the similarity between the noisy patches as described in Subsection 2.2.2 and (2) the probability of the similarity derived from the previous iteration.

Assume that the previous estimation at *t* iteration is $\hat {\theta }^{t-1}$ for *θ* ^∗^. Then, the formula in Eq. 5 can be expressed as:

6 $$ \omega(i,j)^{(\mathrm{It-PPB})}\overset{\bigtriangleup}{=}p\left(\theta_{[v]_{i}}^{*}=\theta_{[v]_{j}}^{*}\mid\upsilon,\hat{\theta}^{t-1}\right)^{\frac{1}{h}}.  $$

This is similar to what was achieved in Subsection 2.2.2, where the probability of the similarity is decomposed into a product of the probabilities of the *k* neighbors: $\prod _{k}p\left (\theta _{i,k}^{*}=\theta _{j,k}^{*}\mid \upsilon _{i,k},\upsilon _{j,k},\hat {\theta }^{t-1}\right)$.

From the Bayesian framework, the naïve Bayes model can be fitted with the maximum likelihood concept. The probability is estimated using the prior probability and can be presumed to be proportional to the likelihood:

7 $$ p\left(\upsilon_{i,k},\upsilon_{j,k},\hat{\theta}^{t-1}\mid\theta_{i,k}^{*}=\theta_{j,k}^{*}\right).  $$

The similarity likelihood is computed using:

8 $$ \begin{array}{ccc} p\left(\theta_{i,k}^{*}=\theta_{j,k}^{*}\mid\upsilon_{i,k},\upsilon_{j,k},\hat{\theta}^{t-1}\right)\propto\\ {\underset{\text{likelihood}}{\underbrace{p\left(\upsilon_{i,k},\upsilon_{j,k}\mid\theta_{i,k}^{*}=\theta_{j,k}^{*}\right)}}\times\underset{\text{prior}}{\underbrace{p\left(\theta_{i,k}^{*}=\theta_{j,k}^{*}\mid\hat{\theta}^{t-1}\right)}}}. \end{array}  $$

The likelihood term is to compute the degree of similarity between the patches, and the prior term is to compare the two probability distributions from the previous iteration, similar to Polzehl and Spokoiny [51].

**Fig. 3:**
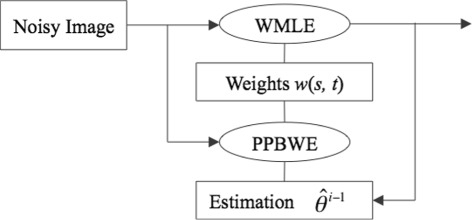
Weights in PPB algorithm: computing iteratively the weights between two pixels *s* and *t* in the probabilistic patch-based (PPB) filter. The PPB weights estimator (PPBWE) uses the noisy image and the estimation values from the previous iteration in order to estimate the weight

The scheme, Fig. 3, shows the procedure of iteratively competing the weights in the PPB algorithm. The procedure for defining the weights is estimated iteratively by (1) the PPB weights estimator (PPBWE) uses the likelihood term and the estimated value from the previous iteration in order to compute the prior term (Eq. 8), (2) WMLE uses the PPBWE estimation and the noisy image in order to estimate the new weight (Eq. 4), and (3) the PPBWE and the WMLE steps are repeated until there is no difference in the estimations made from the two steps.

#### Algorithm used in the case of Gaussian noise

The PPB filter can be used for filtering additive white Gaussian noise (AWGN). By assuming the AWGN model, the values of the pixels *I* of the patch [ *v*]_*i*_ are distributed based on the Gaussian distribution *ℵ*(*u*,*σ* ^2^). Here, *u* is the noiseless image and *σ* the noise variance. The noiseless image *u* can be estimated by the weighted average that maximizes the WMLE defined in Eq. 4:

9 $$ \ddot{u_{i}}^{(\text{WMLE})}=\frac{{\sum\nolimits}_{j}\omega\left(i,j\right)I_{j}^{2}}{{\sum\nolimits}_{j}\omega\left(i,j\right)}.  $$

In order to estimate the weighted average *ω*(*i*,*j*), two terms are considered: the likelihood and the prior terms. The likelihood function is discretized as:

10 $$ \underset{\text{likelihood}}{\underbrace{p\left(I_{i,k},I_{j,k}\mid\ddot{u_{i,k}}=\ddot{u_{j,k}}\right)}}\propto\exp\left(-\frac{\mid I_{i}-I_{j}\mid^{2}}{4\sigma^{2}}\right),  $$

and the prior term is discretized as:

11 $$ \underset{\text{prior}}{\underbrace{p\left(\ddot{u_{i,k}}=\ddot{u_{j,k}}\mid\ddot{u}^{t-1}\right)}}\propto\exp\left(-\frac{1}{T}\frac{\mid\ddot{u}_{i,k}^{t-1}-\ddot{u}_{j,k}^{t-1}\mid^{2}}{\sigma^{2}}\right).  $$

By combining the two terms in Eqs. 10 and 11, the weight at any iteration is defined as:

12 $${} \begin{array}{ccc} \omega(i,j)^{(\text{It}-\text{PPB})}=\\ &{\exp\left[-\underset{n}{\sum}\left(\frac{1}{h}\frac{\mid I_{i}-I_{j}\mid^{2}}{4\sigma^{2}}+\frac{1}{T}\frac{\mid\ddot{u}_{i,k}^{t-1}-\ddot{u}_{j,k}^{t-1}\mid^{2}}{\sigma^{2}}\right)\right]}, \end{array}  $$

where *n* is number of pixels and *T* is a constant similar to *h* in Eq. 2. When there is no iteration “posterior term = 0,” the filter performs similar to the NL-Means filter.

PPB filter has several advantages and disadvantages. The main advantage of PPB filtering is that it is a statistical-based approach, which can be utilized for suppressing additive Gaussian noise and/or multiplicative speckle noise. Also, this filter can be adjusted as an iterative filter or not. Nevertheless, the main drawback of this filter is the suppression of fine and dark details when denoising. Another disadvantage of the filter is the high computational cost when used as an iterative filter.

A further extend patch log likelihood (EPLL) filter, similar to probabilistic patch-based filter, was proposed recently by Papyan and Elad [47] via considering a multi-scale prior.

### Dictionary learning

Dictionary learning (DL) is utilized as a replacement for the use of a fixed dictionary for representing data. From the 1970s, data can be represented by using a fixed dictionary, for instance, Fourier of Boussinesq [5] and Wavelets of Haar [23]. In 1996, Olshausen and Field [45] proposed an approach to learn the dictionary from a data in order to optimize the sparsity of the data. The dictionary learning or k-means singular value decomposition (K-SVD) was first adapted to image denoising in 2006 by Aharon et al. [1]. Dictionary learning method finds the best dictionary $D=\left (d_{i}\right)_{i=1}^{z}$ of *z* atoms $d_{i}\in \mathbb {R}^{n}$ that sparses the set $Y=\left (y_{j}\right)_{j=1}^{m}\in {\mathbb {R}}^{n\times m}$ of signals $y_{j}\in \mathbb {R}^{m}$. In order to filter a noisy image, each signal *y*_*j*_ is considered as a patch extracted from the noisy image. The sparse code of signal data *y*=*y*_*j*_ for *j*=1,…,*n* is obtained by minimizing a constrained optimization *ℓ* ^0^:

13 $$ \underset{\left\Vert x\right\Vert_{0}\leq k}{\min}=\frac{1}{2}\left\Vert y-Dx\right\Vert^{2},  $$

where *k*>0 controls the amount of sparsity, and *ℓ* ^0^ pseudo-norm is defined by:

14 $$ \left\Vert x\right\Vert_{o}=\left\{ i:x_{i}\neq0\right\}.  $$

In dictionary learning, optimization is performed on the dictionary *D* and the coefficients $X=\left (x_{j}\right)_{j=1}^{m}\in \mathbb {R}^{p\times m}$ for *j*=1,…,*n*, where the set of coefficients is *x*_*j*_ of the data *y*_*j*_. The joint optimization is written as:

15 $${} \underset{D\in\Phi,X\in\chi_{k}}{\arg\min}E(X,D)=\frac{1}{2}\left\Vert Y-DX\right\Vert^{2}=\frac{1}{2}\sum\limits_{j=1}^{m}\left\Vert y_{j}-{Dx}_{j}\right\Vert^{2},  $$

where *Φ* is the constraint set:

16 $$ \Phi=\left\{ D\in\mathbb{R}^{n\times p}:\forall i\:\left\Vert D.,i\right\Vert \leq1\right\}.  $$

The sparsity constraint is set on *χ*_*k*_, which is the unit normalization of the dictionary columns:

17 $$ \chi_{k}=\left\{ X\in\mathbb{R}^{p\times m}:\forall i\:\left\Vert X.,i\right\Vert_{0}\leq k\right\}.  $$

Peyré and Fadili [49] proposed using a block-coordinate descent minimization approach used by Tseng [59] in order to minimize *X* and *D*.

The dictionary learning algorithm depends mainly on three steps: (1) patch extraction, (2) sparse coding, and (3) patch construction. In the first step, several patches are randomly extracted from the whole input image. In the sparse coding step, the energies of the *X* and *D* dictionaries are iteratively minimized. Patch averaging and reconstruction occurs in the patch reconstruction step. Each of the three steps has stages:

1. Patch extraction step:
  The mean of each patch is removed from each pixels value.
  Patches are sorted based on their energy, those with a high level of energy are kept by thresholding.
  Patches are reshaped as columns in order to form *Y*.
2. Sparse coding step:
  Each atom in the columns is normalized in order to form the initial dictionary *D*.
  Number of the columns is reduced again, before computing the *X* coefficients.
  The *X* dictionary is initially started with zeros.
  The coefficients of dictionary *X* is updated by:
    $$X=T\left(X-\gamma D'\left(\left(D-X\right)-Y\right)\right), $$
    where *T* is the thresholding.
  The dictionary *D* is updated again by:
    $$D=T\left(D-\gamma\left(\left(DX-X\right)-X'\right)\right). $$
*K* is the number of iterations, which is used to minimize the *X* and *D* dictionaries by updating them iteratively.
  Finally, the coefficients of dictionary *X* are multiplied by dictionary *D*.
3. Patch construction step:
  The result of the multiplication is a new array, *Y*1.
  The columns of *Y*1 are reshaped to form patches.
  Re-inserting the averages to the patches comes before averaging the patches to replace the noisy patch.

**Fig. 4:**
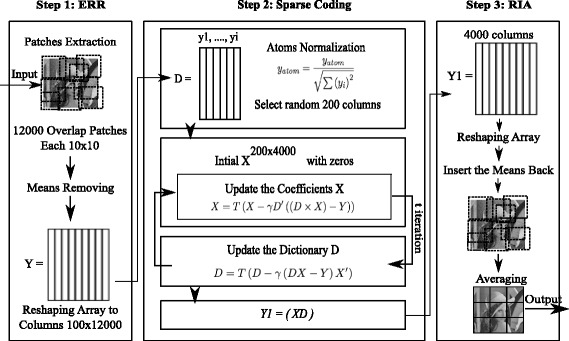
K-SVD filter: dictionary learning scheme of the K-SVD filter

The scheme in Fig. 4 shows the steps of the dictionary learning method for denoising images.

The most important advantage of the DL method is the sparse representation of the input data. One advantage of the DL is the low number of parameters when compared to other patch-based denoising methods. However, DL has some drawbacks. The main disadvantage of DL is the computational burden due to several iterations for computing the singular value decomposition. Moreover, DL does not handle images with many flat patches very well because the singular value decomposition is more sensitive to textured patches.

The DL method has many modifications. Tian and Wang [57] made DL more sparsely representative in the case of less observation values by proposing an adaptive orthogonal matching pursuit to adaptively ensure the sample size. Some of the modifications aim to adapt the idea of denoising based on dictionary learning to other image processing applications. Chen et al. [10] generalized the idea of the learning dictionary to explore identity information in multiple frames of videos. They generated a sparse representation from multiple video frames for face and body part recognitions. Fu et al. [19] proposed an effective model based on DL for hyperspectral image (HSI) denoising by considering sparsity across the spatial-spectral domain, high correlation across spectra, and non-local self-similarity over space. Kang et al. [31] proposed a feature-based approach for assessing similarity between images. After extracting feature points from an image, they utilize dictionary learning. Then, they measured the similarity between images in terms of sparse representation. A novel self-learning based image decomposition framework was presented by Huang et al. [27]. Their framework performs unsupervised clustering on the observed dictionary via affinity propagation that allows effectively to identify images components with similar context information. The framework can automatically determine the undesirable random noisy components from true image components directly from a noisy image. Dictionary learning algorithm was adapted to filter Chinese character images by Shi et al. [54]. They divided the image frequency to low and high frequencies. While a Butterworth low-pass filter was utilized to filter low frequency, the K-SVD dictionary learning algorithm was proposed to filter high-frequency parts which consists of structure of Chinese characters.

### Patch-based PCA

Recently, over-complete dictionaries with sparse representation techniques became very widespread in image denoising [1, 40, 41], and they are one of the state-of-the-art denoising algorithms. These methods use over-complete dictionaries derived from enormous image sets or from the noisy image itself. They outperform other denoising techniques due to their ability to provide an appropriate basis for separating noisy signals from the true image signals, so they suppress more noise and preserve edges. Despite the fact that over-complete dictionaries are frequently used for image denoising, such dictionaries are sophisticated and quite expensive in terms of memory usage and time. However, patch-based principal component analysis (PB-PCA) of Deledalle et al. [13] is a modification of the dictionary methods.

**Fig. 5:**
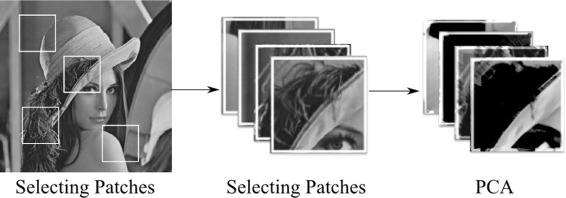
Patches of PB-PCA method: extracting patches in PB-PCA method and grouping them before PCA

PB-PCA uses simple orthogonal dictionaries constructed by using principal component analysis. The results of PB-PCA still do not outperform the over-complete dictionary methods, but PB-PCA shows how simple orthogonal dictionaries can achieve excellent results with less sophistication. PB-PCA simply learns the orthogonal dictionaries from the noisy image via principal component analysis. The next step is to threshold the patches’ coefficients in the dictionaries. This idea is similar to the wavelet denoising methods used in [8, 9, 14], in which they use either hard thresholding or soft thresholding for zeroing the coefficients. Figure 5 shows extracting patches used in the PB-PCA method from an image and grouping them before computing the PCA.

For the problem of denoising an image that is interrupted by AWGN, the patch model has the following formula:

18 $$ [\!v]_{i}=[\!u]_{i}+z_{i},i=1,..,n-1,  $$

where [ *u*]_*i*_ is the true image patch, *z*_*i*_ is the AWGN noise, [ *v*]_*i*_ is the noisy patch, and *n* is the number of patches. By assuming [ *v*]_*i*_,..,[ *v*]_*i*−1_ are a group of overlapped patches of size *N*×*N* extracted from the noisy image *υ*, the covariance matrix is the sum of:

19 $$ \sum=\frac{1}{n}\sum\limits_{i=1}^{n}[\!v]_{i}[\!v]_{i}^{\prime}-\bar{\upsilon}\bar{\upsilon}^{\prime},  $$

where

$$\bar{\upsilon}=\frac{1}{n}\sum\limits_{i=1}^{n}[\!v]_{i}. $$

**Fig. 6:**
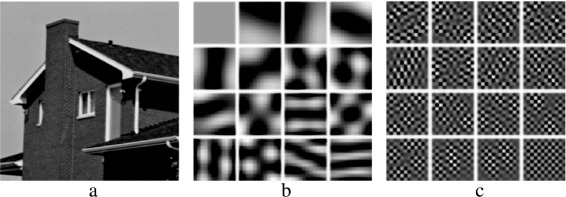
The principal components “axis” of the house image: **a** is the input image, **b** is the first 16 principal axes of the all patches obtained from the house image, and **c** is the last 16 principal axes of the all patches obtained from the house image [13]

In PCA, the singular value decomposition (SVD) of the covariance matrix $\sum $ is processed. Moreover, the eigenvalues *g*_1_,⋯,*g*_*n*−1_ of the covariance matrix and the corresponding eigenvectors *G*_1_,⋯,*G*_*n*−1_ are calculated. Eigenvectors are called the principal components “axis” of the processed data and are used to form an orthogonal basis, *G*_*i*_ is the the *i*th principal axis of the data. Due to the orthogonal basis of the principal components, an image patch can be decomposed as $[\!v]_{i}={\sum \nolimits }_{i=1}^{n}\left \langle [\!v]_{i}\mid G_{i}\right \rangle G_{i}$. Figure 6 b, c shows the first and last 16 principal axes of the all patches obtained from the house image shown in Fig. 6 a.

By assuming that the true image pixels have a low-dimensional subspace and the noise is spread in all directions, projecting the axes into the first axis would suppress the noise in the noisy image. Projecting the axes is called *coefficient thresholding*, and it is done by using an appropriate shrinkage function. A general formula for estimating a true image is:

20 $$ \hat{[\!u]_{i}}=\bar{\upsilon}+\sum\limits_{i=1}^{n}\eta\left(\left\langle [\!v]_{i}-\bar{\upsilon}\mid G_{i}\right\rangle \right)G_{i},  $$

where *η* is the shrinkage function.

**Fig. 7:**
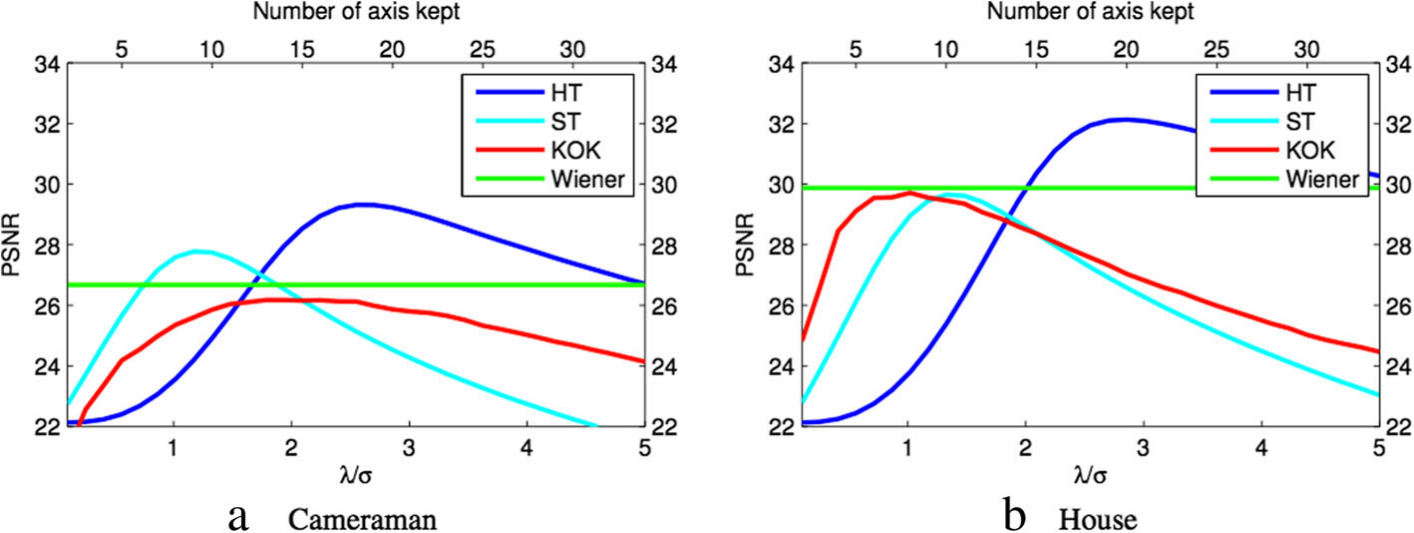
Different PB-PCA projections: a comparison between PSNR of the different methods of the projections in PB-PCA for the House and Cameraman images. The threshold ratio (*λ*/*σ*) into the bottom of *x*-axis controls the number of axes kept in the upper *x*-axis. *σ* is the noise variation, and *λ* is chosen by cross validation [13]

PB-PCA filtering method has been tested with four shrinkage functions: (1) soft thresholding (ST); (2) hard thresholding (HT); (3) Keep or Kill (KoK), and (4) Wiener filter [62]. Figure 7 shows a comparison between the four different projection methods into the basis by PCA for the House and Cameraman images. From Fig. 7, it can be seen that hard thresholding with a large number of axes is the best of the four projection methods. Using a Wiener filter as a shrinkage function for the PCA has been proposed by Muresan and Parks [44, 67]. The Zhang et al. [67] algorithm will be discussed in Subsections 2.4.1.

**Fig. 8:**
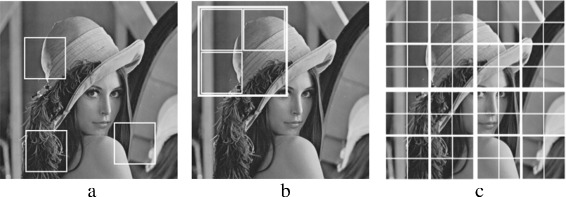
Collecting a set of patches in PB-PCA filtering: **a** global PCA, **b** local PCA, and **c** hierarchical PCA

PB-PCA has three variants based on how each set of patches is collected from the input noisy image before the PCA process. In PB-PCA, the three variants for collecting patches are globally, locally, or hierarchically. Figure 8 shows the best means of collecting the patch sets globally, locally, or hierarchically. These variants are discussed in the following subsections.

#### Patch-based global PCA

Collecting patches for a PCA can be done globally from the entire noisy image in patch-based global PCA (PGPCA). This approach is faster than the other approaches, which need time to divide the image into sub-images before computing the PCA coefficients. However, it has less filtering quality. Collecting patches globally for PCA filtering has been used in Bacchelli and Papi [2] and Zhang et al. [67]. The Bacchelli and Papi algorithm uses a linear transformation, “a wavelet transform,” before computing the PCA in order to achieve better results. The Zhang et al. algorithm collects patches globally, but it computes the PCA in two stages.

The PGPCA approaches cannot compete with other local or semi-local approaches, which consider the high level of redundancy occurring between neighboring patches. In the global PGPCA, one original basis for the whole image, which impacts negatively on the denoising process. The global PGPCA does not identify the rare patches because they do not exert a strong influence on the total variance. However, an allowance can be made for these limitations by considering the local redundancy between patches.

#### Patch-based local PCA

In the patch-based local PCA (PLPCA) approach, patches are collected locally in order to overcome the limitations of the global PGPCA approach. The local collection of patches means that the patches are collected within a small region of interest in the noisy image. A fixed search window *N*×*N* is applied to the whole image. Since the patches are overlapped in PLPCA, there will be multiple estimates for a single pixel. Averaging is used to compute a single pixel’s value.

The advantage of this approach is that the orthonormal basis is adapted only to the sub-image and not to the whole image. However, this approach has two limitations: the overfitting and the fact that it is time-consuming. The overfitting problem is due to the limited number of patches on which to compute the PCA. PLPCA is extremely time-consuming because the PCA needs to be computed repeatedly.

Fei et al. [18] collected patches locally, but they improved their approach by using geometric structure clustering to guarantee that only patches with similar properties were gathered. Pal et al. [46] considered patch redundancy in order to improve on the global two-stage PCA approach of Zhang et al. A sliding window that moves with a step $s=\frac {W_{p}-1}{2}$, where *s* is the step size and *W*_*p*_ is the window’s current location was utilized as a modification in order to reduce the time-consuming element of the PLPCA approach. The computational time is divided by *s* ^2^ without losing the denoising quality. Zhang et al. [68] proposed using similar patch-based local PCA filter with an extended step where a Wiener filter is finally applied.

#### Patch-based hierarchical PCA

In the patch-based hierarchical PCA (PHPCA) approach, an algorithm builds a hierarchy cluster of the patches. Clustering is the task of grouping a set of patches into the same cluster, i.e., set. There are different cluster models; each model has several clustering algorithms. The models include connectivity models (e.g., hierarchical clustering) and centroid models (e.g., k-means). The hierarchical clustering is based on the concept of the grouping of patches according to a maximum distance between patches. Patches are represented as a dendogram, which is a Greek word meaning a tree diagram that illustrates the arrangement of the patches. In centroid models, vectors are assigned usually to a number *k* of fixed clusters. For more information about the clustering models, readers are referred to Chapter 17 in *Introduction to Information Retrieval* by Manning et al. [42].

The objective of the PHPCA approach is to offer a solution that can provide a result between the PGPCA and PLPCA approaches, which is less time-consuming than the local approaches and is more adapted to local sub-images. PHPCA uses a geometric partitioning to first divide the image into four areas, and then it estimates the principal axis for each area. Each area has its own principal components. This process is repeated until the end of the tree is reached. Several dictionaries share the first axes. Figure 8 c shows how an image can be divided to sub-images.

The most important advantage of patch-based PCA filter is its ability to produce a solution for image denoising using a modest orthogonal dictionary with PCA of the input data. Moreover, a patch-based PCA filter provides various grouping stages to fit user needs—local, global, or hierarchical. The main disadvantage of PB-PCA is its susceptibility to overfitting due to few training patches when performing the PCA locally. Also, PB-PCA is quite expensive in terms of time since PCA needs to be performed repeatedly.

### Sparse 3D transform-domain collaborative filtering

**Fig. 9:**
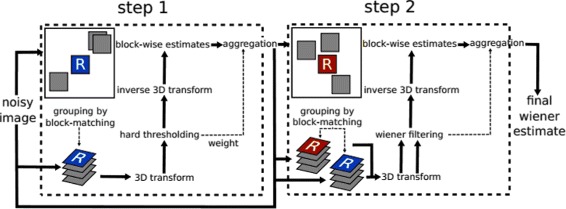
BM3D filtering: the two steps of BM3D filtering [11]

Block matching 3D algorithm (BM3D) is the state-of-the-art denoising technique by Dabov et al. [11]. It is based on modified sparse representation in the frequency domain. BM3D groups the patches into 3D data arrays instead of into 2D arrays, then it applies a modified sparse representation in the frequency domain. Collaborative filtering is used to deal with the 3D arrays. BM3D’s algorithm depends on two steps: (1) collaborative hard thresholding and (2) the collaborative Wiener filtering. The two steps allow the BM3D to suppress more noise and to preserve more detail. The amount of noise is suppressed in the thresholding step, and the details are restored in the second step. Collaborative hard thresholding has three functions: (1) 3D transform, (2) shrinkage, and (3) 3D inverse transform. The patches in the 3D arrays are overlapped, so a weighted average is used to obtain one estimation for each pixel. Aggregation is the averaging procedure. A significant filter is obtained by using the BM3D algorithm. The scheme in Fig. 9 shows the two steps of BM3D filtering. Below, the two steps of the BM3D algorithm are described. First, the collaborative hard thresholding step is explained. Then, using the collaborative Wiener filtering is discussed.

#### Step 1: thresholding

##### Grouping:

Similar to the NL-Means, a search window is used to determine the similarity between the patches. The search window is used in order to benefit from the high redundancy among the neighboring patches. There are several different grouping techniques. A number of these techniques have been discussed in this survey. Some other useful techniques for grouping could be considered for patch grouping, such as vector quantization [21], *k*-means clustering [39], self-organizing maps [34], and others discussed in this survey [29]. However, grouping in BM3D is based on the similarity distance between patches, the “Euclidian distance.” The grouping stage is the first of the two steps in which similar patches are gathered to form 3D arrays. Similarity is computed according to the distances between the patches. Patches with a distance that is below a fixed threshold are considered to be similar and are grouped into the 3D array. Before measuring the distance, a coarse pre-filtering is used to linearly transform the patches using a 2D linear transformation such as multiple wavelet transforms [15, 53]. The formulation in Eq. 21 is used to compute the similarity distance between patches,

21 $$ \begin{array}{c} \\ \text{Dst}\left([\!v]_{i},[\!v]_{j}\right)=\frac{\left\Vert \gamma^{\mathrm{2D}}\left(T_{\text{hard}}^{\mathrm{2D}}\left([v]_{i}\right)\right)-\gamma^{\mathrm{2D}}\left(T_{\text{hard}}^{\mathrm{2D}}\left([v]_{j}\right)\right)\right\Vert_{2}^{2}}{\left(N_{1}^{\text{hard}}\right)^{2}} \end{array},  $$

where [ *v*]_*i*_,[ *v*]_*j*_ are respectively the reference patches at *i* and its neighbors at *j*,$T_{\text {hard}}^{\mathrm {2D}}$ is the 2D linear transform, *γ* ^2D^ is a hard-thresholding operator equals to *λ*_2D_×*σ*, and $\left (N_{1}^{\text {hard}}\right)^{2}$ is the patch size *N*×*N*. *σ* is the estimated noise standard deviation. *γ* ^2D^ makes all coefficients with absolute value less than the threshold (*λ*_2D_×*σ*) equal to zero, and it leaves the other coefficients unchanged. After computing the Euclidian distance, grouping the similar patches into a 3D array is required. The formulation in Eq. 22 is used for gathering similar patches.

22 $$ \mathrm{3D}\,S_{i}^{\text{hard}}=\left\{ j\in\Omega:\text{Dst}\left([\!v]_{i},[\!v]_{j}\right)\leq T_{\text{match}}^{\text{hard}}\right\},  $$

where $\mathrm {3D}\,S_{i}^{\text {hard}}$ is the constructed 3D array contains similar patches and $T_{\text {match}}^{\text {hard}}$ is the maximum distance between two similar patches. The maximum grouped patches size are restricted to $N_{2}^{\text {hard}}$. The next stage is to apply the collaborative filter by (1) performing a 2D linear transform then a 1D linear transform, (2) shrinkage, and (3) inverting the 1D transform and the 2D linear transform.

##### Collaborative filtering:

Once the 3D array is built, a collaborative filter is used for suppressing the noise. A 3D transform is applied to the 3D array, before the shrinkage of the transforming coefficients. The 2D transformation in the grouping stage is applied along both horizontal and vertical lines for each patch, and then a third transformation is conducted along the third diminution of the 3D array for the 3D transform. The formulation of the collaborative filter is:

23 $$ \mathrm{3D}\,\hat{u}_{S_{i}^{\text{hard}}}=T_{\text{hard}}^{\mathrm{3D}^{-1}}\left(\gamma^{\mathrm{3D}}\left(T_{\text{hard}}^{\mathrm{3D}}\left(\mathrm{3D}\,S_{i}^{\text{hard}}\right)\right)\right),  $$

where $T_{\text {hard}}^{\mathrm {3D}}$ is the 3D linear transform of the first (hard) step, $T_{\text {hard}}^{\mathrm {3D}^{-1}}$ is the inverse of 3D transformation, and *γ* ^3D^ is a hard-thresholding operator equals to *λ*_3D_×*σ*. *γ* ^3D^ makes all coefficients with absolute value less than the threshold (*λ*_3D_×*σ*) equal to zero.

##### Aggregation weights:

At this stage, overlapped patches in the 3D array $\left (\mathrm {3D}\,\hat {u}_{S_{i}^{\text {hard}}}\right)$ have multiple estimates for each pixel in the reference patch at the location *i*. A weighted averaging procedure is required to provide an estimate for each pixel. Weights in BM3D are inversely proportional to the total variance of the patches in the $\mathrm {3D}\,\hat {u}_{S_{i}^{\text {hard}}}$ array. When the total variance is high, a small weight is assigned to the patch.

The amount of the additive noise is independent when processing the collaborative filter in step 1 and step 2. Thus, the total variance is not the same after applying the collaborative filter in the first and second steps. In step 1, the total variance is computed by $\sigma ^{2}\times N_{\mathrm {non-zero}}^{\text {hard}}$, where $N_{\mathrm {non-zero}}^{\text {hard}}$ is the number of non-zero coefficients after the hard thresholding. The total variance calculated in step 2 depends on the results of the Wiener filter coefficients, the weights of step 2 will be explained on the facing page. However, the weights for step 1 is equal to:

24 $$ \omega_{i}^{\text{hard}}=\left\{\begin{array}{cc} \begin{array}{c} \frac{1}{\sigma^{2}\times N_{\mathrm{non-zero}}^{\text{hard}}},\\ 1, \end{array} & \begin{array}{c} \text{if}\rightarrow N_{\mathrm{non-zero}}^{\text{hard}}\geq1\\ \text{otherwise} \end{array}\end{array}\right..  $$

#### Step two: Wiener filter coefficients

##### Grouping:

Grouping in the second step is in some ways similar to the grouping in the first step; but here, the power spectrums of the first step are grouped, not just the patches from the noisy image. The same formula is used:

25 $$ \mathrm{3D}\,S_{i}^{\text{Wiener}}=\left\{ j\in\Omega:\frac{\left\Vert \hat{u}_{S_{[v]_{i}}^{\text{hard}}}-\hat{u}_{S_{[v]_{j}}^{\text{hard}}}\right\Vert_{2}^{2}}{\left(N_{1}^{\text{Wiener}}\right)^{2}}\leq T_{\text{match}}^{\text{Wiener}}\right\},  $$

where $\hat {u}_{S_{[v]_{i}}^{\text {hard}}}$ and $\hat {u}_{S_{[v]_{i}}^{\text {hard}}}$ are the estimated sub-images from step 1 respectively at locations *i* and *j*, respectively. At this stage there are two groups: (1) a group of similar patches derived from the noisy image and (2) a group of similar patches derived from the first step.

##### Collaborative filtering:

After grouping the patches, a 3D transform is applied to the 3D array of the grouped patches. A Wiener shrinkage is applied to the transform coefficients of the 3D array. The definition of the Wiener shrinkage coefficients of the power spectrum of the first step is shown in the equation:

26 $$ \mathrm{3D}\,W_{S_{i}^{\text{Wiener}}}=\frac{\left|T_{\mathrm{3D}}^{\text{Wiener}}\left(\mathrm{3D}\,S_{i}^{\text{Wiener}}\right)\right|^{2}}{\left|T_{\mathrm{3D}}^{\text{Wiener}}\left(\mathrm{3D}\,S_{i}^{\text{Wiener}}\right)\right|^{2}+\sigma^{2}},  $$

where $T_{\mathrm {3D}}^{\text {Wiener}}$ is the 3D linear transform and $\mathrm {3D}\,S_{i}^{\text {Wiener}}$ is the result of Eq. 25. The final stage in the collaborative Wiener filtering of the second step is to multiply the Wiener shrinkage coefficients element-by-element by the 3D transform coefficients of the noisy image. The inverse of the 3D transform is applied. Multiplication and the inverse of the 3D transform are shown in the equation:

27 $${} \begin{array}{ccc} \mathrm{3D}\,\hat{u}_{S_{i}^{\text{Wiener}}}=\\ &\!{T_{\mathrm{3D}}^{\mathrm{Wiener^{-1}}}\left(\mathrm{3D}\,W_{S_{i}^{\text{Wiener}}}\times\left(T_{\mathrm{3D}}^{\text{Wiener}}\left(\mathrm{3D}\,\nu_{i}\right)\right)\right)}, \end{array}  $$

where 3D *ν*_*i*_ is the 3D transform coefficients of the noisy data.

##### Aggregation weights:

Adjusting the weights in this step is not like the first step, which depends on the number of non-zero coefficients reached after the hard thresholding. The weights, here, depend on the Wiener shrinkage coefficients; the weights are assigned as:

28 $$ \omega_{i}^{\text{Wiener}}=\sigma^{-2}\left\Vert W_{S_{i}^{\text{Wiener}}}\right\Vert_{2}^{-2}.  $$

The parameters set for the original BM3D is shown in Table 1; the table has some non-discussed parameters:

- $N_{\mathrm {step1}}^{\text {hard}}$: step size for searching the patches inside the search window

**Table 1:** Parameters set for the original BM3D

	Symbol	Description	Fast BM3D	Normal BM3D	
				*σ*≤40	*σ*≥40
Step 1 (hard) parameters	$T_{\text {hard}}^{\mathrm {2D}}$	2D transform	Bior1.5	Bior1.5	DCT
	$T_{\text {hard}}^{\mathrm {3D}}$	3D transform	Haar	Haar	Haar
	$N_{1}^{\text {hard}}$	Patch size	8	8	12
	$N_{2}^{\text {hard}}$	3D array size	16	16	16
	$N_{\mathrm {step1}}^{\text {hard}}$	Patch step	6	3	4
	$N_{\text {search}}^{\text {hard}}$	Window size	25	39	39
	$N_{\mathrm {step2}}^{\text {hard}}$	Window step	6	1	1
	$N_{\mathrm {Prev.}}^{\text {hard}}$	Small window	3	–	–
	*β* ^hard^	Kaiser window	2.0	2.0	2.0
	*λ*_2D_	2D thresholding	0	0	2.0
	*λ*_3D_	3D thresholding	2.7	2.7	2.8
	$T_{\text {match}}^{\text {hard}}$	Distance	2500	2500	5000
Step 2 (Wiener) parameters	$T_{\text {Wiener}}^{\mathrm {2D}}$	2D transform	DCT	DCT	DCT
	$T_{\text {Wiener}}^{\mathrm {3D}}$	3D transform	Haar	Haar	Haar
	$N_{1}^{\text {Wiener}}$	Patch size	8	8	11
	$N_{2}^{\text {Wiener}}$	3D array size	16	32	32
	$N_{\mathrm {step1}}^{\text {Wiener}}$	Patch step	5	3	6
	$N_{\text {search}}^{\text {Wiener}}$	Window size	25	39	39
	$N_{\mathrm {step2}}^{\text {Wiener}}$	Window step	5	1	1
	$N_{\mathrm {Prev.}}^{\text {Wiener}}$	Small window	2	–	–
	*β* ^Wiener^	Kaiser window	2.0	2.0	2.0
	$T_{\text {match}}^{\text {Wiener}}$	Distance	400	400	3500
- $N_{\mathrm {step2}}^{\text {hard}}$: step size for moving the search window
- $N_{\mathrm {Prev.}}^{\text {hard}}$: small search window width for fast BM3D
- *β* ^hard^: the Kaiser window function one parameter for reducing the borders effect
- Haar: a Haar transform
- DCT: a discrete cosine transform
- Bior1.5: a biorthogonal wavelet

BM3D filter has advantages and disadvantages. The main advantage of BM3D filtering is that it yields great results with less loss of detail due to the smoothing and sharping stages. The BM3D filter is a fast method because it computes similarity between patches before the actual filtering procedure. Despite its many advantages, BM3D has several drawbacks. The performance of BM3D decreases with a high noise level (*σ*>40), and it produces images with many artifacts. BM3D is more complex and less flexible to be adapted for domain-specific image processing applications. In addition, it is not easy to be parallelized. In addition, it has many parameters, and adjusting them optimally is a challenging task.

BM3D has two filtering steps and more than 20 parameters. Improving the way of adjusting any of the 20 parameters would participate on improving the output of the BM3D method. BM3D modifications would be categorized into four main categorizes: (1) modifications consider improving the shrinkage function, (2) modifications consider improving the transforms, (3) modifications consider improving the image similarity measures when collecting similar patches, and (4) modifications consider improving the Wiener filtering stages. Suwabe et al. [55] modified the way of collecting similar patches in the BM3D from non-locally to globally. They proposed using iterative filtering with Chebyshev polynomial approximation (CPA) in order to collect the patches from the whole noisy image. Bashar and El-Sakka [3] replaced the fixed hard thresholding scheme with a learning-based adaptive hard thresholding scheme that considers the context of corresponding blocks. Hasan and El-Sakka [24] improved the Wiener filter of BM3D by maximizing the structural similarity (SSIM) [60] between patches instead of using the mean square error (MSE). Moreover, they introduced a 3D zigzag thresholding.

## Results and discussions

**Fig. 10:**
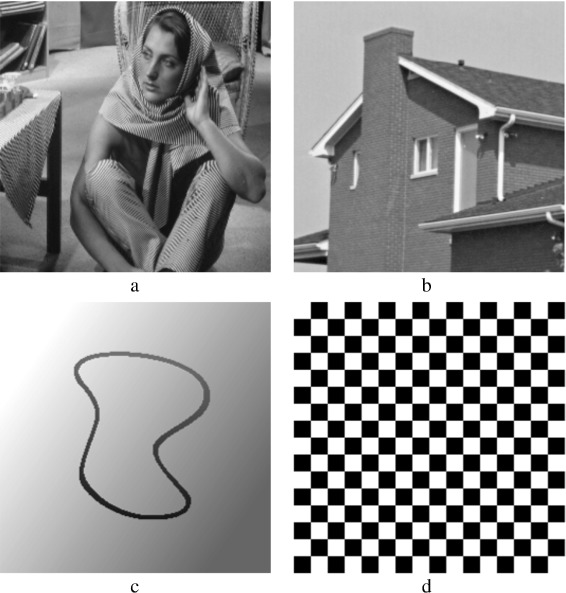
The four used images in the experiment: **a** *Barbara* image 512×512, **b** *House* image 256×256, **c** *CurvedBand* image 257×257, and **d** *Chessboard* image 256×256

In this section, various denoising methods are compared aiming to reduce additive white Gaussian noise (AWGN). The objective of this section is to experimentally study the performance of these methods, where the performance is assessed at various noise levels. The issue of time consumption is also addressed. Four images are used to run this experiment. The images have been chosen carefully to help in distinguishing between the methods. The first two of the four images are natural scene images, Barbara and House; the other two are synthetic images, CurvedBand and Chessboard. The Chessboard image is a binary image while the other three images are gray-scale images. The four images are shown in Fig. 10. The fine details in Barbara image helps in demonstrating how various methods preserve the image clarity, whereas the sharp edges in the House image helps in demonstrating how various methods preserve edges. The gray gradations in CurvedBand image provide insight into the amount of smoothing that has been applied to images. The methods are also tested with the binary pattern repetitions in the Chessboard image. MatLab is used for this experiment. The computer’s processor is an Intel (R) Core(TM) i7 CPU @ 3.40 GHz. In Subsections 3.1 and 3.2, the methods are evaluated both quantitatively and qualitatively.

### Quantitative evaluation

In order to make an objective comparison between the results, two image similarity matrices are used: (1) the structural similarity and (2) the peak signal-to-noise ratio (PSNR). These quality metrics have been chosen for their capability to assess the level of the additive Gaussian noise degradation. The best result for SSIM is 1, while the PSNR has good result when its value is high. Equations 29 and 30 show the formulas for these two quality metrics, respectively:

29 $$ \text{SSIM}(x,y)=\frac{\left(2\mu_{x}\mu_{y}+C_{1}\right)\left(2\sigma_{xy}+C_{2}\right)}{\left(\mu_{x}^{2}+\mu_{y}^{2}+C_{1}\right)\left(\sigma_{x}^{2}+\sigma_{y}^{2}+C_{2}\right)},  $$

where *x* is the true reference image, *y* is the noisy image, *μ*_*x*_ and *μ*_*y*_ are the mean of the true reference image block and the noisy image block, respectively, *σ*_*x*_ and *σ*_*y*_ are the variance/covariance of the true reference image block and the noisy image block, respectively, and *C*_1_ and *C*_2_ are constants used to avoid instability. The peak signal-to-noise ratio is defined as:

30 $$ \text{PSNR}=10 \ \text{log}\left(\frac{\left(2^{n}-1\right)^{2}}{\text{MSE}}\right),  $$

where MSE is the mean squared error and *n* is an integer number representing the number of bits per pixel. When *n*=8, i.e., in case of gray-scale images, the PSNR formula is reduced to:

31 $$ \text{PSNR}=10\ \text{log}\left(\frac{255^{2}}{\text{MSE}}\right).  $$

A study conducted by Hore and Ziou [26] has revealed that SSIM is less sensitive to additive noise than PSNR. They used *F*-score test to compare between SSIM and PSNR which works for AWGN. Thus, the final conclusion in our study is driven based on SSIM.

The experimental results of the denoising methods are shown in Tables 2 and 3. The tables show the performance of the patch-based denoising methods along with the famous pixel-based denoising methods: anisotropic diffusion (AD) by Perona and Malik [48] and the bilateral filter. In both tables, the methods are sorted from the oldest to the most recent.

**Table 2:** The performance of the denoising algorithms with various noise levels (*σ*)

Bold font indicates the best performance

**Table 3:** Execution time in seconds shows the speed of the denoising methods applied to the four images at various noise levels (*σ*)

Bold font indicates the best performance

The results in Table 2 are computed by measuring the differences between the original images and the denoised images. The default parameters shown in Table 1 were used for the BM3D method, these values are suggested by the authors of BM3D. The noise standard deviation of the noisy images, which methods depends heavily on it, is required to be adjusted accurately before the denoising process. In case the noise estimation was not given, Ghazi and Erdogan [22], Tai and Yang [56], and Liu et al. [37] offer more information about noise estimation. The highest values of SSIM and PSNR are highlighted with a bold font. Table 3 shows the execution time of the methods in seconds. The fastest patch-based methods are highlighted with a bold font.

The performance of the denoising methods varies depending on the noise level and the scene details inside noisy images. For example, the pixel-based bilateral filter outperforms the famous patch-based NL-Means method when denoising the Chessboard image at *σ*≤10 because of the large number of flat regions. Using pixel-based methods is not recommended when standard deviation of a noisy image is above 40. Although promising results have been achieved by BM3D when denoising flat and textures scene images at high noise levels *σ*>10, the performance of BM3D decreases when denoising images have a lot of fine details regions at *σ*≤10; see the results of Barbara and House images in Table 2.

**Fig. 11:**
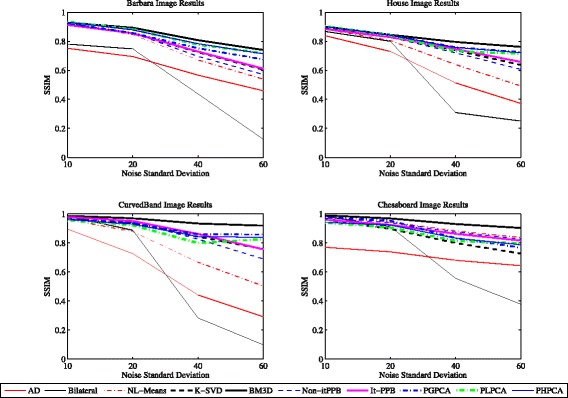
Denoising methods performance: the performance of the denoising methods for the four images at various noise levels (*σ*)

Figure 11 shows the performance of the methods with various levels of noise on each of the four images. The charts show that BM3D is the best method (from SSIM point of view) when the noise level is high. BM3D is the best whether it is used for denoising natural scene or synthetic images. PCA patch-based methods come second after BM3D when they are used for natural scene images. The results of K-SVD and the iterative PPB are similar.

The two lines at the bottom of each chart presents the performance of pixel-wise methods: anisotropic diffusion and the bilateral filter. From the charts, we can conclude that the block-wise denoising methods perform better than the pixel-wise methods. When using *σ*=10 and *σ*=20, the results are similar except with the pixel-wise methods. The performance of the methods will be discussed in the following two paragraphs.

**Fig. 12:**
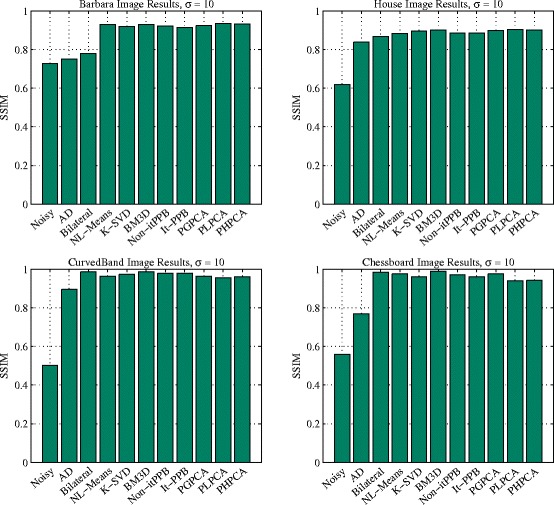
The performance charts: four charts summarize the performance of the denoising methods for the four images when the noise is low (*σ*=10)

Bar charts are used in Fig. 12 to represent the methods performance when the noise level is low *σ*=10. The charts show that patch-based denoising methods are similar. The pixel-wise denoising methods perform better when denoising the synthetic images due to its flat areas and the absent of details. In contrast, the patch-based denoising methods achieved better results when denoising the natural scene images. When the noise level is low *σ*=10, the top two denoising methods for denoising the natural scene images are PHPCA and BM3D, whereas the top two denoising methods for denoising the synthetic images are BM3D and bilateral filter. Yet, the differences are insignificant.

**Fig. 13:**
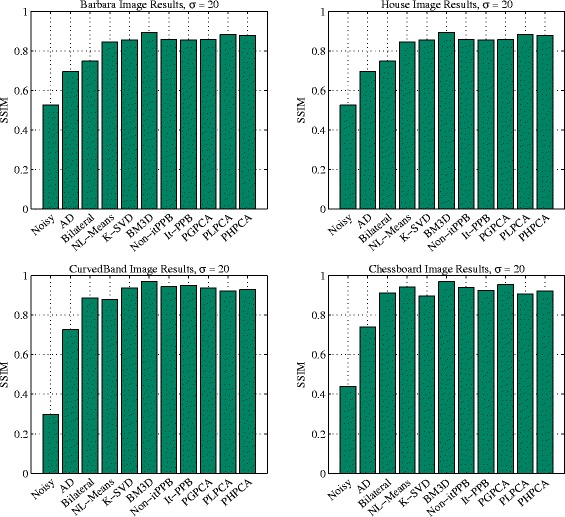
The performance charts: four charts summarize the performance of the denoising methods for the four images when the noise is low (*σ*=20)

The charts in Fig. 13 show the methods performance when *σ*=20. By increasing the noise level, the contrast between methods becomes obvious unlike when the noise is low *σ*=10. BM3D achieved better results than other methods in all images. The patch-based denoising methods perform better than pixel-wise methods.

**Fig. 14:**
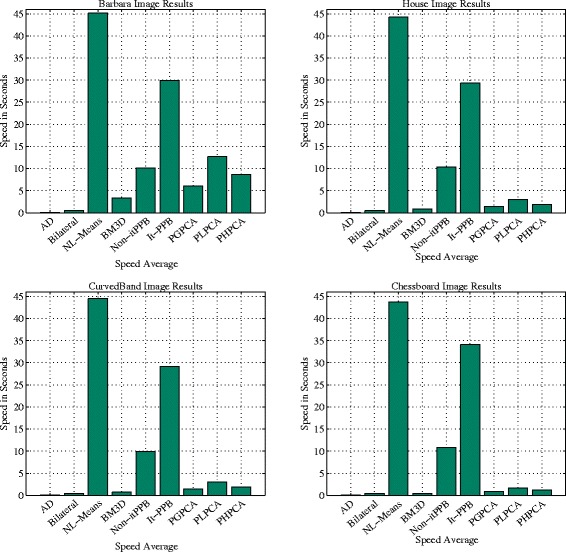
The efficiency charts: four charts show the average of the consumed time in seconds for various denoising methods excluding K-SVD

Figure 14 contains charts that illustrate the average execution time aspect for each image. The K-SVD methods are very time-consuming. They are about ten times as expensive as any other methods. Thus, K-SVD is excluded from the charts to make it easier to distinguish between methods. The NL-Mean comes second after the K-SVD. Although, the BM3D uses two stages to perform the denoising step, it is the fastest patch-based method. The time consumed for various values of noise is almost the same, so the high level of noise does not greatly affect the time consumption of the various methods.

### Qualitative evaluation

**Fig. 15:**
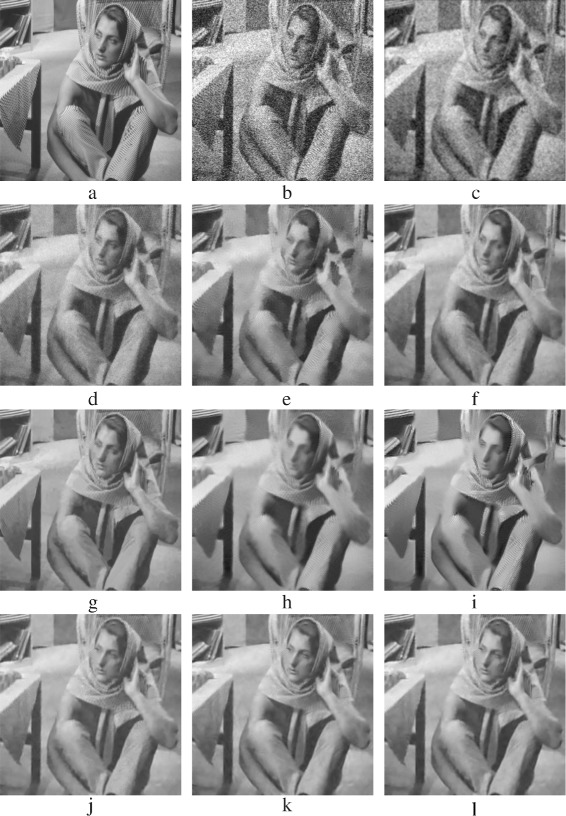
Denoised *Barbara* images: **a** Original *Barbara* image; **b** AWG noise, *σ*=40; **c** AD ; **d** bilateral filtering, **e** NL-Means filtering, **f** K-SVD, **g** BM3D, **h** non-itPPB, **i** it-PPB, **j** PGPCA, **k** PLPCA, and **l** PHPCA

The evaluation in this subsection is a subjective evaluation, where the quality of the denoised images is addressed via the visual perception. Denoised images with AWGN (*σ*=40) are chosen to perform this evaluating. Figures 15, 18, 19, and 21 show the denoised images.

**Fig. 16:**
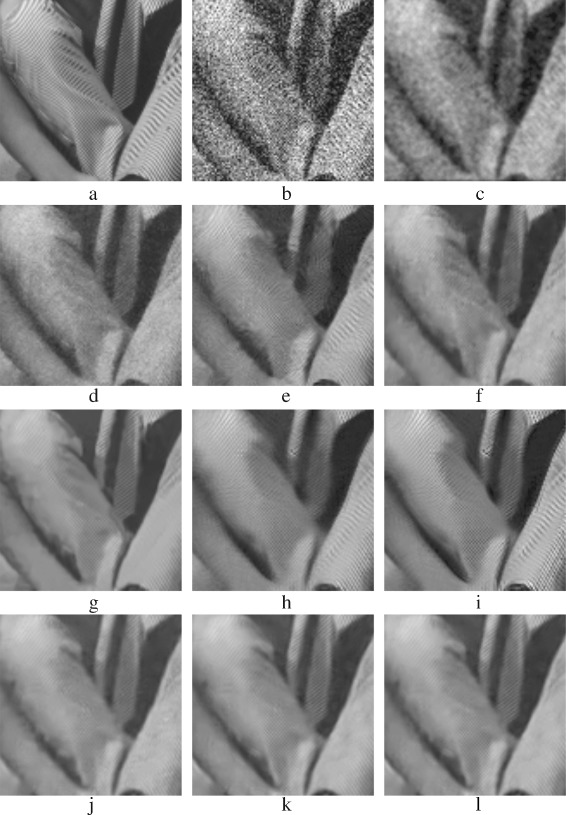
Zoomed images of the denoised *Barbara* image shown in Fig. 15: **a** original Barbara’s pant; **b** AWG noise, *σ*=40; **c** AD (Perona & Malik); **d** bilateral filtering; **e** NL-Means filtering; **f** K-SVD; **g** BM3D; **h** Non-itPPB; **i** It-PPB; **j** PGPCA; **k** PLPCA; and **l** PHPCA

**Fig. 17:**
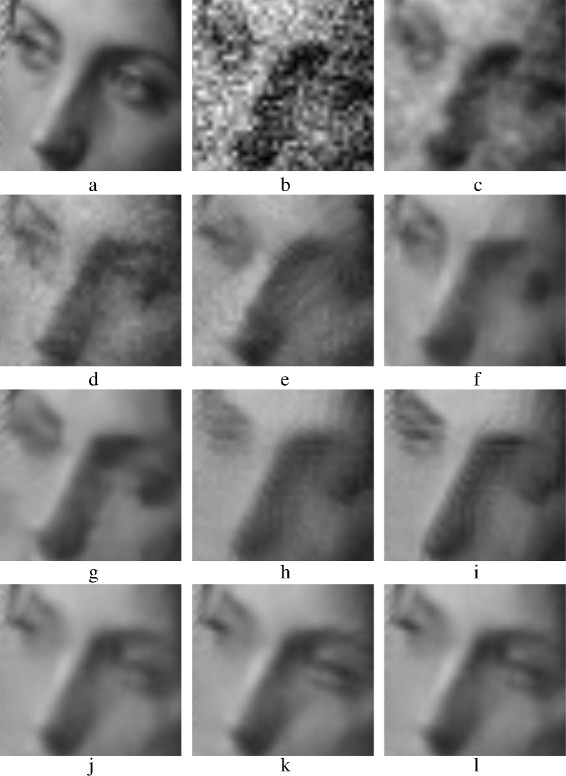
Zoomed images of the denoised *Barbara* image shown in Fig. 15: **a** original Barbara’s eye cover fold; **b** AWG noise, *σ*=40; **c** AD (Perona & Malik); **d** bilateral filtering; **e** NL-Means filtering; **f** K-SVD; **g** BM3D; **h** Non-itPPB; **i** It-PPB; **j** PGPCA; **k** PLPCA; and **l** PHPCA

The results of denoising Barbara’s image are shown in Fig. 15. Figure 15 g shows the best achieved result by using BM3D method. While homogeneous regions are properly smoothed in BM3D, the sharp edges are preserved. The K-SVD and the PCA patch-based methods come after BM3D, Fig. 15 f, j, k, l shows K-SVD and PCA patch-based results. They apply a good smoothing to the image, but some edges are destroyed. The non-local mean method applies less smoothing; thus, the textures shown in Fig. 15 e in Barbara’s pants are preserved; a zoomed version of Fig. 15 e is shown in Fig. 16 e. Figure 15 h, i shows how the PPB methods apply a good smoothing, but they fail to preserve sharp edges and textures, i.e., Barbara’s eye cover fold. Zoomed versions of Fig. 15 h, i are shown in Fig. 17 h, i.

**Fig. 18:**
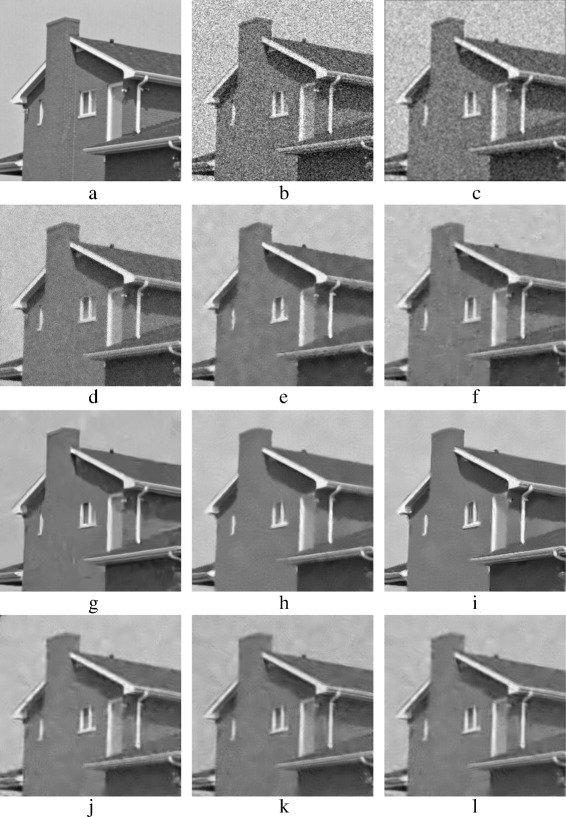
Denoised *House* images: **a** original *House* image; **b** AWG noise, *σ*=40; **c** AD (Perona & Malik); **d** bilateral filtering; **e** NL-Means filtering; **f** K-SVD; **g** BM3D; **h** Non-itPPB; **i** It-PPB; **j** PGPCA; **k** PLPCA; and **l** PHPCA

Figure 18 shows the results of denoising the House image. The best results are achieved by using BM3D and PPB methods, Fig. 18 g–i shows these results. K-SVD and PLPCA methods have disappointing results, unlike their results when they are used for denoising Barbara image. Figure 18 f, k show K-SVD and PCA methods results. The edges are preserved when using K-SVD, but K-SVD fails to smooth properly flat areas, i.e., the sky in the House image. Thin edges are not preserved with PCA patch-based methods.

**Fig. 19:**
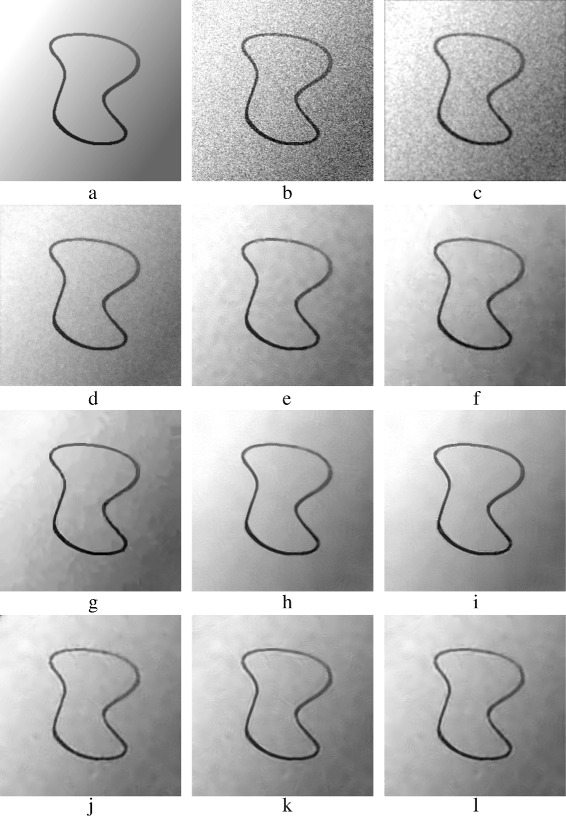
Denoised *CurvedBand* images: **a** original *CurvedBand* image; **b** AWG noise, *σ*=40; **c** AD (Perona & Malik); **d** bilateral filtering; **e** NL-Means filtering; **f** K-SVD; **g** BM3D; **h** Non-itPPB; **i** It-PPB; **j** PGPCA; **k** PLPCA; and **l** PHPCA

**Fig. 20:**
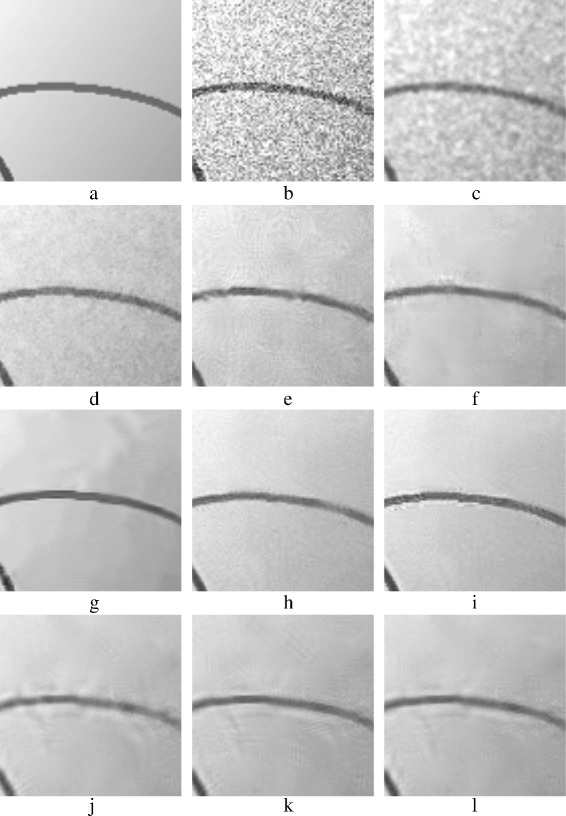
Zoomed images of the denoised *CurvedBand* image shown in Fig. 19: **a** original *CurvedBand* image; **b** AWG noise, *σ*=40**c** AD (Perona & Malik); **d** bilateral filtering; **e** NL-Means filtering; **f** K-SVD; **g** BM3D; **h** Non-itPPB; **i** It-PPB; **j** PGPCA; **k** PLPCA; and **l** PHPCA

Figure 19 shows the denoised CurvedBand image. PPB methods and BM3D have the best results, the results are shown in Fig. 19 g– i. Unlike BM3D, PPB methods succeed more in smoothing the gray gradations; zoomed versions of Fig. 19 g–i are shown in Fig. 20 g–i. K-SVD and PCA patch-based methods have similar results, Fig. 19 f, j, k, l shows these results. They preserve parts of the curve, but they do not smooth properly flat areas; zoomed versions of Fig. 19 f, j, k, l are shown in Fig. 20 f, j, k, l.

**Fig. 21:**
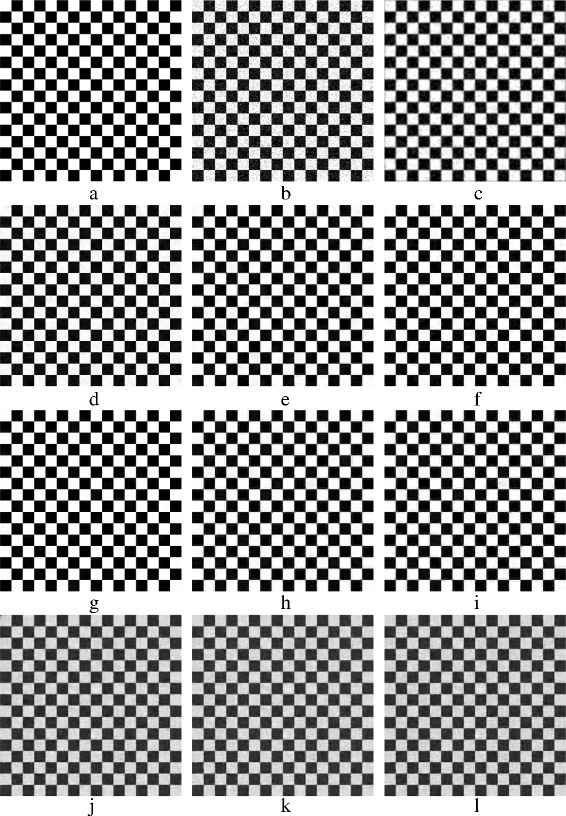
Denoised *Chessboard* images: **a** original *Chessboard* image; **b** AWG noise, *σ*=40; **c** AD (Perona & Malik); **d** bilateral filtering; **e** NL-Means filtering; **f** K-SVD; **g** BM3D; **h** Non-itPPB; **i** It-PPB; **j** PGPCA; **k** PLPCA; and **l** PHPCA

Figure 21 shows the denoised Chessboard image. Figure 21 g shows BM3D result, BM3D achieves the best result when denoising the binary Chessboard’s image. NL-Means method has the second good result, unlike its performance on the other images. Figure 21 e shows the result of NL-Means method. With the K-SVD method shown in Fig. 21 f, edges are preserved and the flat areas are smoothed appropriately. Disappointed results are achieved by using PCA patch-based methods; Fig. 21 j, k, l shows the result of using PCA patch-based methods.

## Summary of contribution

This paper provides a review of the state-of-art patch-based denoising techniques and compares their effectiveness for denoising natural, synthetic, and binary images. Effort is drawn on details like smoothing flat regions and objects and preserving details such as edges, lines, and textures. The features, strengths, and limitations of the patch-based denoising techniques are also presented. Moreover, the review covers the issue of time. Finally, based on years of experience, we believe that this review will be helpful for researchers to choose suitable denoising techniques to be adapted further for their image processing applications.

## Future research directions

Like any other image denoising approaches, many important research directions should remain in patch-based image denoising. Improving patch similarity measures is suggested for grouping accurately similar patches. Moreover, the research should also produce tools with better shrinkage functions to suppress noise and preserve fine details. Finally, developing effective image transform strategies that meet the needs to differentiate between a true and noisy signal is strongly recommended.

## Conclusions

Among the best denoising methods is patch-based denoising method, which includes BM3D, NL-Means, and K-SVD. This paper has dealt with the efficiency of each of these methods when compared to other patch-based denoising methods. Experimentally, BM3D method gives the best result; it performs very well on all images and at all levels of noise. K-SVD and PCA methods come second to BM3D. Although the sparsity is learned from the data itself in K-SVD, K-SVD fails to compete with BM3D. NL-Means gives an encouraging result at low levels of noise. Furthermore, PPB methods preserve the fine details but fails to do so with respect to the sharp edges.

K-SVD is incomparable with the other compared methods in terms of its time consumption. As it is very expensive. In contrast, BM3D execution time is the best among the patch-based denoising methods because it computes all similarity between whole patches first before starting the actual denoising process.

## Abbreviations

AD: Anisotropic diffusion
AWGN: Additive white Gaussian noise
BM3D: Block matching 3D algorithm
CPA: Chebyshev polynomial approximation
DL: Dictionary learning
EPLL: Extend patch log likelihood
HSI: Hyperspectral image
HT: Hard thresholding
It-PPB: Iterative probabilistic patch-based
K-SVD: K-means singular value decomposition
KoK: Keep or Kill
MLE: Maximum likelihood estimator
MSE: Mean square error
MSSIM: Mean structural similarity
NL-Means: Non-local means
NSERC: Natural sciences and engineering research council of Canada
Non-iPPB: Non-iterative probabilistic patch-based
PB-PCA: Patch-based principal component analysis
PCA: Principal components analysis
PGPCA: Patch-based global PCA
PHPCA: Patch-based hierarchical PCA
PLPCA: Patch-based local PCA
POL-SAR: Polarimetric synthetic aperture radar
PPB: Probabilistic patch-based
PPBWE: PPB weights Estimator
PSNR: Peak signal-to-noise ratio
SNR: Signal-to-noise ratio
SSIM: Structural similarity
ST: Soft thresholding
SVD: Singular value decomposition
WMLE: Weighted maximum likelihood estimation
XNL-Means: X-ray non-local means
